# Burning Questions: What Should the Pharmacist Know about Managing Minor Burns?

**DOI:** 10.3390/pharmacy10040100

**Published:** 2022-08-22

**Authors:** Shelley Wall, Velisha Ann Perumal-Pillay

**Affiliations:** 1 Pietermaritzburg Burn Service, Nelson R. Mandela School of Medicine, University of KwaZulu Natal, Private Bag X54001, Durban 4000, South Africa; 2 Discipline of Pharmaceutical Sciences, College of Health Sciences, University of KwaZulu-Natal, Private Bag X54001, Durban 4000, South Africa

**Keywords:** burns, wound care, primary healthcare, pharmacy

## Abstract

Burn injuries are an endemic health concern in developing countries. Globally, Africa has the highest incidence of burn injuries per capita. A total of 2.3% of the South African population suffer burn injuries annually and may present at community pharmacies and primary healthcare (PHC) facilities. Pharmacists and PHC nurses must, therefore, remain abreast with the latest treatments for burn care. This commentary presents the most recent information for assessing burn wounds, first aid, referral guidelines, and identifying toxic shock syndrome in more severe burns cases. The successful management of patients with burns in an outpatient setting is contingent on patient selection. It is important for pharmacists and PHC nurses to know when to treat or refer a patient. Therefore, a set of guidelines for their use in PHC and community pharmacy settings is presented. Appropriate training on the use of these guidelines, would ensure a better assessment of burn wounds, leading to more positive patient outcomes. This commentary is a useful update to continuing professional development and can be utilised in community pharmacies and PHC settings in South Africa and across the African continent in the absence of formalised treatment guidelines for minor burns.

## 1. Introduction

Low- and middle-income countries (LMICs) constitute 70% of burn injuries and 90% of burn fatalities worldwide [1]. Globally, Africa has the highest incidence of burn injuries per capita [2]. Access to burn care in LMIC’s is limited by the lack of adequately trained staff, appropriate equipment and resources [1].

Approximately 3.2% of the South African population sustain burn injuries annually [2]. More than 1.5 million of these are minor injuries [2]. Patients with these injuries may present to primary health care (PHC) facilities or pharmacies seeking advice or treatment. Pharmacists must maintain professional competence with the latest developments in healthcare related to their scope of practice [3], such as burn care.

This paper provides an understanding of the current standard of care for the treatment of burns, leading to the appropriate and rational use of medicines for improved patient outcomes. This is a useful update to the continuing professional development of pharmacists and PHC workers.

## 2. History Taking

Pharmacists should take the history of the patient that can enable a proper assessment of the situation [3]. Information regarding the time of the injury, injury mechanisms and first aid measures must be ascertained. It is imperative to determine if the wound is new, or if there are any concerning associated symptoms that potentially indicate sepsis.

## 3. First Aid

First aid is the first step in burn management. This involves cooling the burn wound with cool running tap water [4]. First aid is effective up to 3 h post-burn and a 20-min duration is most beneficial [4]. First aid cooling reduces thermal spread, burn depth and time to re-epithelialization [4]. Other substances used as first aid can be detrimental to the wound and should be avoided. Burnshield^®^ is commonly used on burns, and while it was previously recommended for first aid, the more recent literature has shown better outcomes for cool water in randomized studies than other, previously recommended cooling methods [5]. Burnshield^®^ is an acceptable alternative for first aid when there is no clean water available [5]. Burnshield^®^ is not a definitive dressing and, if used as part of first aid, needs to be removed and dressed with something definitive once the wound is cooled [6].

## 4. Wound Assessment

A pharmacist’s ability to adequately assess a burn wound is integral when determining if a burn requires referral to a higher medical facility. Burn wound assessment involves assessing both the size (total body surface area (TBSA)) and the depth of the burn. The size of the burn can be determined using various charts. While the Rule of Nines and Lund–Browder charts are useful in larger burns, determining the TBSA of small or patchy burns is difficult with these charts [7]. In these situations, it is easier to use the palmer rule to determine the size of the burn. The palm, including the fingers, of the patient represent 1% TBSA of their body.

It is important to determine the burn depth. Superficial burns can be managed as an outpatient, but all deep burns need to be referred for further management. Superficial burns and superficial partial thickness burns that do not meet other criteria for referral can be safely managed by a pharmacist. Superficial burns are dry, red and blanch with pressure. Superficial partial burns usually have blisters; they are moist, pink and blanch with pressure [8]. Burn depth assessment is further explained in Table 1.

## 5. Who to Refer

The successful management of patients with burns in an outpatient setting is contingent on patient selection [8]. There are many situations where referral to a higher level of care is mandated. The South African Burn Society published a list of minimum criteria for referral to a burn centre [9]. These published criteria address referral to a burn centre, but does not provide guidance regarding who should be referred to a higher level of care when there is no specialized burns unit in a system with high patient pressure on referral hospitals.

From experience as a burn surgeon, being consulted on all burns’ patients due to a lack of knowledge regarding the appropriateness of when to treat or refer, a knowledge gap was evident. There was a need for guidelines to be made available to pharmacists and PHC nurses in such situations. To address this gap, a brief set of guidelines (Box 1) (adapted from the South African Burn Society Minimum Criteria for Referral to a Burn Centre) is presented for use in PHC and community pharmacy settings. If pharmacists and PHC nurses are appropriately trained on the use of these guidelines, they would be better equipped to provide a better judgement and perform a better assessment of burn wounds, to ensure more positive patient outcomes. In these incidences, it is advisable for the pharmacist to provide first aid and, eventually cover the wound with a temporary dressing such as a burn shield or paraffin gauze, and then refer the patient to a higher centre to ensure more positive patient outcomes. Patients with certain co-morbidities are at increased risk of complications. Patients with diabetes may have altered wound-healing and should be referred if the wound does not heal, even if the wound is minor and otherwise does not meet the referral criteria. Burn wounds may become complicated in patients with pre-existing peripheral vascular disease. These patients mandate early referral to higher care.

Box 1. **Burns requiring referral to a higher level of care (adapted from the South African Burn Society Minimum Criteria for Referral to a Burn Centre) [9].**

Superficial burns more than 10% TBSA

 

Deep burns of any size

 

Extremes of age (under 2 years old and over 60 years), fragile patients with few physiological reserves, unless the burn is exceptionally small and superficial

 

Electrical Burns

 

Chemical Burns

 

Deep burns to special areas: face, hands, feet, perineum, large joints

 

Patients with co-morbidities

 

Any sign of burn wound cellulitis or infection: peri-wound erythema and increased temperature.

 

Any burn, assumed to be superficial partial that has not healed in 21 days

 

Any size burn when the patient has any systemic signs of sepsis

 

Any burn with other associated trauma

 

Suspected non-accidental injury

 

Suspected inhalation injury

## 6. Wound Care and Dressings

With the range of dressings available for wound care, it is important for the pharmacist to know how to select an appropriate dressing for a particular wound and how to prevent wound infection.

There is no place for prophylactic systemic antibiotics in acute burn care [6]. The infection prevention strategy lies in wound care in the form of wound cleansing and topical antimicrobial dressings, when available. All wounds and surrounding skin should be cleaned on presentation with chlorhexidine gluconate soapy water to remove debris and blisters, and before subsequent dressings [6].

For many years, silver sulfadiazine was associated with burn care; however, newer studies have shown delayed wound-healing, along with its use in comparison to other modern wound-care options [10]. Silver sulfadiazine requires dressing twice daily, which are not practical, and should be avoided if possible [10].

Many modern wound-care options are available, which provide antimicrobial activity and can be kept in situ for longer periods. In the case of superficial burns, some modern dressings can be left in situ until the wound heals, effectively reducing the pain associated with dressing changes and reducing the healing time by minimizing disruptions to the healing wound with multiple dressings [10]. These include Dialkylcarbomoyl chloride (DACC) containing dressings or bismuth containing dressings, and hydrofiber dressings with silver and silver-containing foams, to name a few. These primary dressings can be left in situ, with the secondary dressings (gauze and crepe bandages) being removed after 3–7 days to ensure the primary dressing is dry and adherent to the wound. If the primary dressing is wet, it needs to be removed and replaced with a new primary dressing to prevent superimposed infection. No modern wound-care technology has been proven to be superior to another [6]. Paraffin gauze is acceptable if nothing else is available; however, daily dressings are then required [6]. Occlusive dressings often result in the pooling of exudate and, as a result, the proliferation of bacteria and wound infection [6].

## 7. Conclusions

The majority of burns are minor and can be managed by pharmacists with appropriate dressings and advice. Thus, pharmacists need to be abreast of the newest recommendations regarding burn assessment and wound care. The information presented here can be utilised by other healthcare professionals in PHC settings in South Africa and across the African continent in the absence of formalised treatment guidelines for minor burns.

## Figures and Tables

**Table 1:** Burns Depth Assessment.

	Epidermal Burn	Superficial Dermal/Superficial Partial Burn	Deep Dermal/Deep Partial Burn	Full Thickness Burn
Pathology	Involves epidermis only	Involves the epidermis and upper dermis	Involves epidermis and most of the dermis	Involves the epidermis and all of the dermis
Appearance	Red only	Pale pink; blanches to pressure	Red or pale pink; non-blanching	Waxy and white; leathery; non-blanching
Blistering	None or only a few days later	Yes—within hours of the burn	Yes—early blistering	No

## Data Availability

Not applicable.
